# Immediate remote ischemic postconditioning after hypoxia ischemia in piglets protects cerebral white matter but not grey matter

**DOI:** 10.1177/0271678X15608862

**Published:** 2015-10-08

**Authors:** Mojgan Ezzati, Alan Bainbridge, Kevin D Broad, Go Kawano, Aaron Oliver-Taylor, Eridan Rocha-Ferreira, Daniel Alonso-Alconada, Igor Fierens, Jamshid Rostami, K Jane Hassell, Ilias Tachtsidis, Pierre Gressens, Mariya Hristova, Kate Bennett, Sophie Lebon, Bobbi Fleiss, Derek Yellon, Derek J Hausenloy, Xavier Golay, Nicola J Robertson

**Affiliations:** 1 Institute for Women’s Health, University College London, London, UK; 2 Physics and Bioengineering, University College London NHS Trust, London, UK; 3 Medical Physics and Biomedical Engineering, University College London, London, UK; 4 Department of Perinatal Imaging and Health, Division of Imaging Sciences and Biomedical Engineering, King’s College London, King’s Health Partners, St. Thomas’ Hospital, London, UK; 5 Inserm, U1141, Paris, France; 6 University Paris Diderot, Sorbonne Paris Cité, Paris, France; 7 PremUP, Paris, France; 8 The Hatter Cardiovascular Institute, University College London, London, UK; 9 Cardiovascular and Metabolic Disorders Program, Duke-NUS Graduate Medical School, Singapore, Singapore; 10 National Heart Centre Singapore, Singapore; 11 Institute of Neurology, University College London, London, UK

**Keywords:** Birth asphyxia, hypoxia–ischemia, neuroprotection, neonatal encephalopathy, remote ischemic postconditioning

## Abstract

Remote ischemic postconditioning (RIPostC) is a promising therapeutic intervention whereby brief episodes of ischemia/reperfusion of one organ (limb) mitigate damage in another organ (brain) that has experienced severe hypoxia-ischemia. Our aim was to assess whether RIPostC is protective following cerebral hypoxia-ischemia in a piglet model of neonatal encephalopathy (NE) using magnetic resonance spectroscopy (MRS) biomarkers and immunohistochemistry. After hypoxia-ischemia (HI), 16 Large White female newborn piglets were randomized to: (i) no intervention (*n* = 8); (ii) RIPostC – with four, 10-min cycles of bilateral lower limb ischemia/reperfusion immediately after HI (*n* = 8). RIPostC reduced the hypoxic-ischemic-induced increase in white matter proton MRS lactate/N acetyl aspartate (*p* = 0.005) and increased whole brain phosphorus-31 MRS ATP (*p* = 0.039) over the 48 h after HI. Cell death was reduced with RIPostC in the periventricular white matter (*p* = 0.03), internal capsule (*p* = 0.002) and corpus callosum (*p* = 0.021); there was reduced microglial activation in corpus callosum (*p* = 0.001) and more surviving oligodendrocytes in corpus callosum (*p* = 0.029) and periventricular white matter (*p* = 0.001). Changes in gene expression were detected in the white matter at 48 h, including K_ATP_ channel and endothelin A receptor. Immediate RIPostC is a potentially safe and promising brain protective therapy for babies with NE with protection in white but not grey matter.

## Introduction

Intrapartum-related hypoxic ischemic events are a significant global burden with 1.15 million babies each year developing neonatal encephalopathy (NE).^1^ The majority (96%) of NE occurs in low and middle-income settings, where mortality and neuroimpairment rates are high.^1^ In high-income settings where neonatal intensive care of a high quality is routinely available, therapeutic hypothermia is a safe and standard practice for term infants diagnosed with NE.^2^ There is clear evidence that therapeutic hypothermia in this setting reduces adverse outcome at 18 months of age (typical RR 0.75, confidence interval: 0.68–0.83)^2^; this improvement persists into childhood resulting in widespread benefit to society, individuals and the economy.^3^ However, therapeutic hypothermia is only partially effective as a neuroprotective therapy for NE as over 45% of infants have adverse neurodevelopmental outcomes despite treatment. Moreover, effective cooling treatment requires a high level of neonatal intensive care support, which is not available in many lower-resource settings. There is an urgent need to develop additional simple, safe and effective neuroprotective treatment strategies that can be used alone or with hypothermia.

Organisms have evolved mechanisms to protect against tissue damage and to compensate and regenerate after injury. There is a growing interest in harnessing endogenous protective strategies to protect the brain from reperfusion injury.^4^ Ischemic preconditioning is a powerful innate protective mechanism against ischemia-reperfusion injury that has evolved in mammalian species. Ischemic preconditioning describes brief non-cell-lethal episodes of ischemia which confer protection against a subsequent sustained period of severe ischemia in the same organ and was first described in the myocardium in 1986.^5^ Ischemic postconditioning (IPostC) evolved from this concept^6^ and is defined as intermittent interruptions in blood flow at the time of reperfusion *after* the severe ischemia in the same organ.^7^ IPostC has an obvious therapeutic timing advantage over preconditioning and the benefits of IPostC appear to be mediated by similar molecular pathways.^8^ In adult rodent models of stroke, IPostC reduced cerebral infarct size in focal^7^ and global ischemia^9^ and was effective if performed on a non-vital organ, such as the limb, remote to the affected organ – *remote IPostC (RIPostC).*^10^ RIPostC can be immediate (applied within a few minutes of reperfusion) or delayed (applied hours to weeks after reperfusion). Ren and colleagues have shown that both immediate and delayed RIPostC provide protection in adult rats and improve neurologic function two months after the ischemic insult.^10^ In neonatal rodent stroke models, reduced infarct volumes and improved neurological outcomes were observed with four 10-min cycles of ischemia and reperfusion of both hindlimbs started immediately after HI.^11^ Even with a 24-h delay after HI in the start of RIPostC, there were improved sensory motor outcomes five weeks after the ischemic insult, although the infarct size was not reduced.^12^

The objective of this study was to assess whether immediate RIPostC is protective following a global cerebral hypoxic ischemic insult in a piglet model of perinatal asphyxia. This model has strong similarities to newborn infants with NE in terms of the timing of the evolution of injury after hypoxia-ischemia (HI),^13,14^ the pattern of injury, neuropathology and cerebral magnetic resonance spectroscopy (MRS).^15^ In addition, meticulous intensive care monitoring and support of the piglet maintain physiological and metabolic stability. RIPostC treatment efficacy was explored using clinically relevant biomarkers of *in vivo* proton (^1^H) MRS for lactate, N-acetyl aspartate (Naa), creatine^16^ and phosphorus-31 (^31^P) MRS for inorganic phosphate, phosphocreatine and NTP.^13^ At 48 h after HI, histological assessment of cell death was performed using TUNEL, cell-specific labeling to measure glial number (S100B and Oligo2), microglial activation (Iba1), and activation of endothelial cells (eNOS). Underlying putative neuroprotective mechanisms were investigated with exploratory genome microarray and qualitative reverse transcription polymerase reaction (qRT-PCR).

## Materials and methods

### Sample size calculation

Our primary outcomes were cerebral lactate/Naa and NTP/exchangeable phosphate pool (epp). Previous work with our model suggested that the change in lactate/Naa during 48 h varied between normo- and hypothermic groups by 1.0U, with a standard deviation of 0.75 U (both log scale). Assuming similar magnitude of additional effect for RIPostC following HI versus HI alone and with 5% significance and 80% power, eight subjects were required in each group.

### Animal experiments and surgical preparation

**Figure 1.**
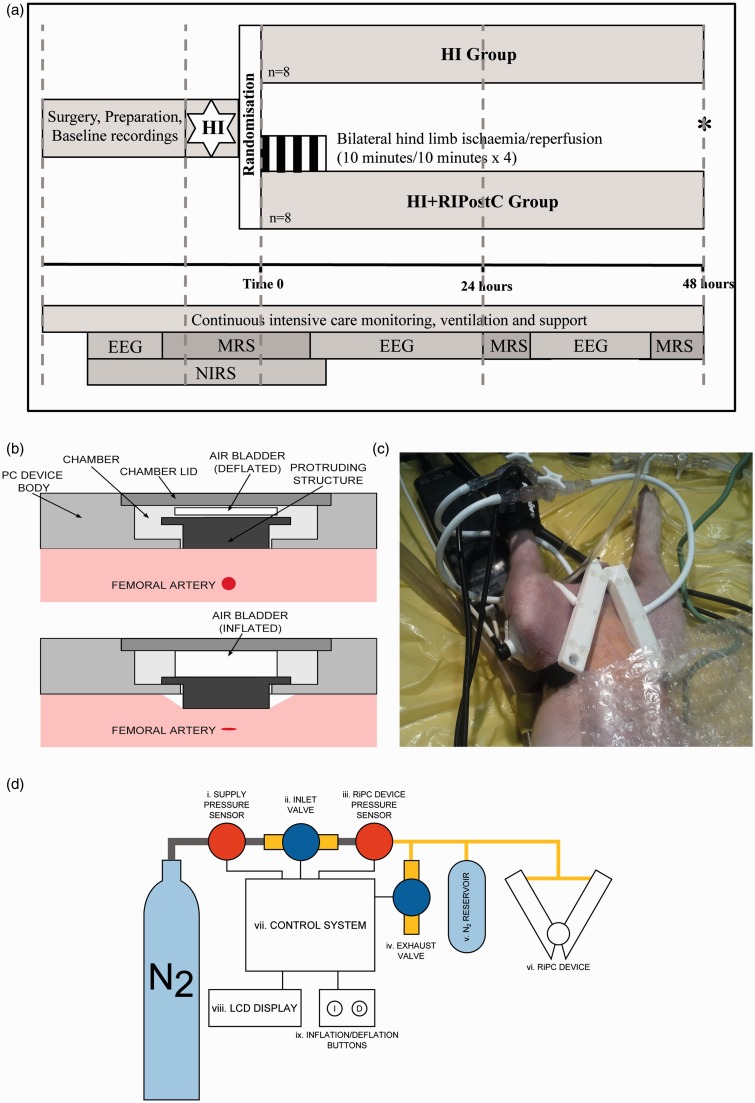
(a). Study time-line. Following baseline data acquisition, piglets underwent cerebral hypoxia-ischemia. At the end of hypoxia-ischemia (time 0), piglets were randomized to (i) HI or (ii) HI + RIPostC. RIPostC was started immediately at Time 0. Piglets (Continued) **Figure 1**. Continue. were maintained under meticulous intensive care for 48 h at which point (*) they were euthanized. MRS was acquired at baseline, during HI, for the first 60 min after HI, and at 24 and 48 h. NIRS was acquired at baseline, during HI and throughout the four RIPostC cycles. EEG was acquired at baseline and in between the MRS acquisitions. (b) Side view of the RIPostC device remotely controlled in the bore of the 9.4 Tesla MR system. The top panel shows the device during the reperfusion period with the occluder in its resting position. The lower panel shows the inflated air bladder pushing the occluder forward to compress the femoral artery lying below. (c) The occluders are shown here with occlusion of both femoral arteries inducing lower limb ischemia. The lower limbs are clearly cyanosed. Limb ischemia was further confirmed by loss of the signal from the pulse oximeter and laser doppler in both limbs. (d) Diagram showing the complete RIPostC system used to remotely occlude both femoral arteries in the magnet. The closed system was connected to a nitrogen gas cylinder; pressure sensors allowed the gas pressure to be measured. The device was controlled by the inflation buttons, which allowed gas to enter the system, inflate the bladders, and occlude the femoral arteries.

All animal experiments were approved by the Ethics Committee of University College London and performed according to the UK Home Office Guidelines [Animals (Scientific procedures) Act, 1986]. The study complies with the ARRIVE guidelines. Sixteen female piglets, aged less than 30 h, with a weight range of 1.6–2.1 kg were anesthetized and surgically prepared as described previously.^14^ The study time-line is shown in Figure 1(a). Studies were started around 9 am and all studies were performed in the same laboratory. Following initial assessment for any signs of obvious infection including diarrhea and conjunctivitis, an intramuscular dose of 0.2 mg/kg of midazolam was administered for sedation. Anesthesia was induced by 4% v/v isoflurane through a facemask for around 5 min to facilitate tracheostomy and intubation. Throughout the surgery, isoflurane was maintained at 2.8–3% guided by peripheral oxygen saturation monitoring (Nonin Medical, Plymouth, MN, USA) and the animal’s response to stimulation. Following tracheostomy, a suitable size of endotracheal tube (Smiths Medical, Ashford, Kent, UK) was fixed and the piglet was mechanically ventilated (SLE 2000 infant ventilator, Surrey, UK). Ventilator settings were adjusted to maintain partial pressure of oxygen (PaO_2_) at 8–13 kPa and carbon dioxide (PaCO_2_) at 4.5–6.5 kPa, allowing for temperature and fraction of inspired oxygen (FiO_2_) correction of the arterial blood sample.

After the airway was secured, both common carotid arteries were surgically isolated at the level of the fourth cervical vertebra and a vascular occluder (OC2A, In Vivo Metric, Healdsburg, CA, USA) was placed on each side. After completion of surgery, the inspired isoflurane concentration was maintained at 2% v/v.

An umbilical venous catheter was inserted for infusion of maintenance fluids (10% dextrose, 60 ml/kg/day before the insult and 40 ml/kg/day after resuscitation), fentanyl (5 µg/kg/h), and antibiotics (benzyl penicillin 50 mg/kg, every 12 h and gentamicin 4 mg/kg, once a day). An umbilical arterial catheter was inserted for invasive physiologic monitoring (SA instruments) for heart rate and arterial blood pressure, and blood sampling for arterial gases and electrolytes (Abbot Laboratories, UK). Hepsal (0.5 IU/ml of heparin in 0.9% saline solution) was infused at rate of 0.3 ml/h to prevent umbilical arterial catheter blockage.

After surgery, and before securing piglets in the custom-built holder, the postconditioning device was placed over both inguinal canals and strapped diagonally across the inguinal region overlying an inflatable bladder. To assess limb perfusion during and after RIPostC, a separate pulse oximeter was attached to right hind limb, while laser Doppler assessed perfusion on the left side. An additional pulse oximeter was attached to right forelimb to monitor the systemic oxygen saturation.

Piglets were cared for under intensive care conditions throughout the experiment. To maintain the MABP above 40 mmHg, bolus infusions of 0.9% saline (Baxter; 10 ml/kg), dopamine (5–20 µg/kg/min), dobutamine (5–20 µg/kg/min) and adrenaline (0.1–1.5 µg/kg/min) were used as required by a NICU trained clinician. High serum lactate was treated by optimizing oxygenation and administering 0.45% saline bolus infusions. Hyperkalemia (K > 7.0 mmol/l) was treated with 4 mcg/kg salbutamol (10 mcg/ml) through the umbilical venous catheter over 10 min. Calcium gluconate (0.5 ml/kg of 10% solution) was administered slowly to stablize the myocardium when ECG changes or hypocalcemia (ionized calcium <1 mmol/l) occurred. In the presence of metabolic acidosis, a half correction of sodium bicarbonate was given after ensuring adequate ventilation.

### MR methods

The head was immobilized in a stereotactic frame for MRS acquisition. Piglets were positioned within the bore of 9.4 Tesla Agilent MR scanner. ^1^H and ^31^P MRS spectra were acquired at baseline and at 24 and 48 h after cerebral HI, described below.

#### ^31^P MRS

A 7 cm × 5 cm elliptical transmit-receive MRS surface coil tuned to the ^31^P resonant frequency (51.6 MHz) was positioned on top of the head. ^31^P MRS was acquired with 1-min resolution using a non-localized single-pulse surface-coil acquisition (repetition time 10 s, six summed acquisitions per spectrum). MRS data were analyzed using AMARES^17^ as implemented in the jMRUI software. Prior knowledge of NTP multiplet structure was used (fitting doublets to α- and γ-NTP and a triplet to β-NTP), but no assumption was made as to multiplet relative sizes. NTP is predominately ATP and the latter contributes approximately 70% of the NTP signal.^18^ Thus, NTP changes during this experiment predominately reflected ATP changes. Pi was fitted using four separate components and PCr with a single component. The following peak-area ratios were calculated: Pi/epp, PCr/epp, and NTP/epp where epp = exchangeable phosphate pool = Pi + PCr + 2γ-NTP + β-NTP.

#### ^1^H MRS

^1^H MRS data were collected from voxels located in the dorsal right subcortical white matter at the centrum semiovale level (white matter voxel, 8 × 8 × 15 mm) and in the deep grey matter centered on both lateral thalami (deep grey matter voxel, 15 × 15 × 10 mm) using a combination of a 65 × 55 mm elliptical receive surface coil, a 150 mm transmit volume coil and a LASER acquisition (TR = 5000 ms, TE = 288 ms, 128 averages). Spectra were analyzed using AMARES as implemented in the jMRUI software and the lactate/Naa peak ratio was calculated.

#### Statistics

Analyses were carried out using Stata 13. MRS data were analyzed by fitting a mixed effects linear regression model with a random subject effect included in the model for each metabolite ratio. The variance–covariance structure of these models allows for the fact that two observations from an individual subject are more likely to be similar than are observations from two different subjects. Measurement time points are referred to as baseline, 24 h post HI and 48 h post HI. Fitting an interaction term between the time and the treatment group allowed different regression slopes for each time and group combination to be estimated.

### Cerebral hypoxia–ischemia (HI)

NIRS and ^31^P MRS were acquired continuously for 10 min at baseline, during HI, and for 1 h after cessation of HI. HI was induced inside the MR scanner by remotely inflating the vascular occluders around both common-carotid arteries, and simultaneously reducing FiO_2_ to 6% (vol/vol). During HI, the β-NTP peak height was continuously monitored using in-house Matlab (Mathworks) software. At the point at which β-NTP had fallen to 50% of its baseline value, FiO_2_ was increased to 9%. When β-NTP fell to 40% baseline height, the inspired oxygen fraction was titrated to keep the β-NTP peak height between 30% and 40% of its original height for a period of 12.5 min. At the end of HI, the carotid arteries were de-occluded and the FiO_2_ returned to 21%. Insult severity was estimated by calculating the time integral of the change in NTP/epp during HI and the first 60 minutes of resuscitation, as described previously.^19^

### Remote ischemic postconditioning method/device

We designed a purpose built RIPostC device, suited to piglet anatomy. Computer-aided design software was used (Autodesk Inventor Professional 2013) to 3D print the device (Selective Laser Sintering) in nylon. The V-shaped device was secured by Velcro straps directly over the inguinal creases and femoral arteries. Occlusion of the femoral arteries leading to bilateral limb ischemia was induced by inflation of the air bladder within the device; this pushed the occluder downwards out of the device to compress the femoral artery lying below (Figure 1b and c). Reperfusion was induced by deflation of the air bladder within the device; this allowed the occluder to return back into the device. This was performed remotely by a controller (Figure 1d), which consisted of two solenoid valves (2 port VDW10 series, SMC Pneumatics LTD, Crownhill, UK), one to gate the inflow of high-pressure nitrogen for inflation, and the second as an exhaust to control deflation. The pressure in the air bladder was measured using an electronic sensor (MPX5050, Freescale Semiconductor, Arizona, USA) and monitored continuously using a microcontroller (Arduino Uno). During each 10-min inflation, the bladders were maintained at a pressure of at least 40 kPa. A LCD display was used to provide visual feedback of the bladder pressure, and display the elapsed time since the last inflation/deflation. An analogue voltage display proportional to the pressure within the PC device, and a digital gating signal (high when inflated) were shown on the front panel for monitoring and recording.

### Experimental groups

Following resuscitation, while in the bore of the MR system, piglets were randomized (computer generated randomization revealed after HI to minimize bias) into two groups – HI or HI with remote ischemic postconditioning (HI + RIPostC) (Figure 1a). There were eight animals in each group. In the HI group, the postconditioning device was fitted but not inflated. In the HI + RIPostC group, immediately after resuscitation, piglets underwent four cycles of 10-min ischemia followed by 10-min reperfusion in both hindlimbs using the RIPostC device. Limb ischemia was confirmed by laser Doppler velocimetry in the left hindlimb and pulse oximetry of the right hindlimb. Both groups were cared for over 48 h after HI and maintained normothermic (38–38.5℃). Physiological parameters were compared between groups with Mann–Whitney at each time point.

### Electroencephalography and amplitude-integrated electroencephalography

A six lead EEG was acquired between MRI studies. aEEG background voltage activity was classified according to al Naqueeb et al.^20^ Grade 3 was assigned to normal voltage (upper margin > 10 μV, lower margin > 5 μV); Grade 2 to moderately abnormal voltage (upper margin > 10 μV, lower margin ≤ 5 μV) and Grade 1 assigned to severely abnormal aEEG (upper margin < 10μV, lower margin < 5 μV). aEEG grading was performed at baseline (before insult), 3, 6, 12, 24, 36 and 48 hours post insult. An ordered logistic regression model was fitted to the data, allowing for repeated measurements over time in each pig to estimate the odds ratio (OR) of change in aEEG with RIPostC.

### Brain histology

Piglets were euthanized by pentobarbital injected at 48 h after the HI insult and the brain fixed via cardiac perfusion with cold PBS, followed by 4% paraformaldehyde in PBS. After nine days post-fixation in 2% paraformaldehyde, 5 mm thick coronal slices of the right hemisphere from the optic chiasma were processed to paraffin wax, sectioned (8 µm) and stained with hematoxylin and eosin to validate the bregma for analysis. For each animal, two sections (bregma 00 and −2.0) were stained (described below) and 12 different regions in the brain were examined.

TUNEL staining for DNA fragmentation and immunohistochemistry (IHC) were performed as previously described.^21^ In brief, TUNEL sections were pre-treated in 3% hydrogen peroxide, subjected to a protease K pre-digestion (Promega, Southampton, UK) and incubated with TUNEL solution (Roche, Burgess Hill, UK). TUNEL was visualized using avidin-biotinylated horseradish complex (ABC, Vector Laboratories, Peterborough, UK) and diaminobenzidine/H_2_O_2_ (DAB, Sigma, Poole, UK) enhanced with CoSO_4_ and NiCl_2_. For IHC, sections were rehydrated, heat treatment used for antigen retrieval and following blocking with appropriate serum together with 0.1 Triton in PBS, sections were incubated with primary antibody overnight at 4 ℃: Iba1 (1:1000; Wako, Osaka, Japan); S100 (1:800, Abcam, Cambridge, UK); Olig2 (1:400, Millipore, Nottingham, UK); eNOS (1:1000, BD Biosciences, Oxford, UK). Sections were incubated with a biotinylated secondary antibody (1:250) and staining was visualized using ABC (both Vector Laboratories) and DAB (Sigma). TUNEL and IHC sections were dehydrated and cover-slipped with DPX (VWR, Leighton Buzzard, UK).

Investigators blind to the treatment groups performed the analyses. Counts of TUNEL and Olig2 positive cells were made in three non-overlapping fields of view for each brain region at two levels at ×40 magnification (0.15 mm^2^ per field). The activation state of Iba-1 positive cells was assessed by scoring the process number, process complexity (primary, secondary and tertiary), and relative intensity of the soma staining from 0 to 4 (score 0, thin process with tertiary branches and able to visualize the cresyl violet counterstain through the Iba-1 of the soma; score 1, tertiary processes but increased staining intensity of the soma; score 2, reduced numbers of tertiary processes and thickening of secondary processes with intense enlarged soma; score 3, loss of secondary processes and short thickened primary with intense soma; score 4, no process and intensely stained soma). Scores were taken of four cells per FOV at ×20 magnification (at set positions from the corners of the image) for a total of 24 cells per region per animal. Two investigators, blinded to treatment group, scored each image independently and their scores were averaged. The levels of astroglial activation and endothelial cell nitric oxide synthase expression were assessed via S100B and eNOS immunoreactivity, respectively. Mean and standard deviation of optical luminosity values were measured in 3 non-overlapping fields (×20 magnification) of the different brain regions of eNOS and S100B stained slides using Optimas 6.5 image software. Standard deviation was subtracted from the mean of each field and the resulting value was subtracted from the value obtained from the surround glass.

For each of the outcomes, TUNEL, Iba-1, S100B, Olig 2 and eNOS separate linear mixed effects models were fitted (using log transformed data for the S100B and TUNEL outcomes to satisfy normality assumptions) in order to investigate the effect of RIPostC after HI compared to no treatment. Including a random subject effect in the model allowed for correlations between observations from the same subject that arise from repeated measurements, as each outcome is measured in several fields in each area of the brain. It is assumed that the correlation between any two observations from the same subject is constant, and that observations from different subjects are independent. For each outcome, estimation of the fixed treatment effect was carried out using restricted maximum likelihood in order to obtain unbiased estimates from the small sample sizes, and was tested using a Wald test. The Olig2 was analyzed using Poisson mixed effects models with a random subject effect as above.

### Gene expression analysis

#### RNA extraction

After euthanasia, before perfusion, a sample of white matter from the opposite hemisphere to that used for immunohistochemistry was obtained and placed in RNAlater solution (Qiagen, West Sussex, UK), frozen in liquid nitrogen and stored at −80 ℃ until processing. RNA was extracted using the standard protocol for animal tissues supplied with the RNAeasy Midi kit (Qiagen, West Sussex, UK). RNA quality was assessed using a Nanodrop spectrophotometer (NanoDrop, Wilmington, DE, USA) and Agilent 2100 Bioanalyser (Agilent, Santa Clara, CA, USA) and all samples had a spectral 260/280 ratio of between 2.05 and 2.13, and a RNA Integrity number of 9.9–10.

#### RNA amplification and microarray hybridization

For each sample, 200 ng of total RNA was amplified and labeled using an Ambion WT expression kit (Invitrogen, Life Technologies Ltd, Paisley, UK). Briefly, total RNA was converted to cDNA and then linearly amplified to create an antisense cRNA library. This was then converted to single strand sense cDNA, which was fragmented and end-labeled before hybridization using a Gene Chip WT terminal labeling and controls kit (Affymetrix, California, USA). The amplified targets were hybridized to Gene Chip Porcine Genome Arrays (Affymetrix, California, USA) overnight and scanned using Gene-Chip Scanner 3000 7 G. Data files were extracted from the image files automatically by Gene-Chip Command Software (version 2, Affymetrix, California, USA) and the CEL file format was subsequently used for analysis.

#### Microarray analysis

Analysis was performed using Genespring GX12 (Agilent, California, USA). Data from the individual microarray chips was first normalized across dataset and summarized using the Robust Multi-array Analysis (RMA) algorithm. Data were filtered to include only those probe sets falling between the 20th and 100th percentile after normalization.

Initial analysis with Genespring GX12 probe sets detected a total of 74 gene transcript sets. We identified unknown probe sets using Basic Local Alignment Search Tool (BLAST) and BLAST-Like Alignment Tool (BLAT) sequence alignment software against the pre-labeled mouse genome. Ingenuity Pathway Analysis software (IPA; Ingenuity Systems, California, USA) was used to assign the 74 identified genes to a range of known biological functions and metabolic or signaling pathways. Statistical analysis of microarray data was performed using a one-way ANOVA followed by a Mann–Whitney unpaired post hoc test and a Benjamini-Hochberg FDR multiple testing correction. *P*-values were calculated asymptotically.

#### Quantitative reverse transcription polymerase chain reaction

RNA isolated for microarray analysis was also used for qualitative reverse transcription polymerase reaction (qRT-PCR). Sample preparations, primer design, and PCR protocol were similar to that previously described.^22^ Primers were designed specifically using the Sus scrofa Ensembl database and can be found in Supplementary Table 1. The reference genes 14-3-3 protein zeta/delta (YWAHZ) and ribosomal protein L4 (Rpl4) were chosen to standardize all quantitative experiments. For each duplicate sample, we averaged the calculated specific ratio of the gene of interest/reference gene. Statistical analysis of the qRT-PCR data was performed by T test or Mann Whitney depending on whether the data were normally distributed; *p* < 0.05 was considered as significant.

## Results

### Physiological data and Insult severity

There were no statistically significant intergroup differences between body weight, postnatal age and baseline physiological (heart rate and mean arterial blood pressure) measures as shown in Table 1. There was no significant difference in the hypoxic-ischemic insult severity between the two groups (Table 1). There was no difference between groups for volume replacement and inotrope use following HI (Table 2).

**Table 1.** Physiological variables and blood pressure treatment for piglets in each group.

Parameter	HI Mean (SD)	HI & RIPostC Mean (SD)	*p*-value
Post-natal age (h)	35.8 (10.9)	33.0 (10.0)	0.279
Bodyweight (kg)	1.82 (0.22)	1.83 (0.21)	0.645
Duration of HI (min)	20.5 (2.6)	20.6 (1.9)	0.594
Insult severity (10^−2^) measured from acute energy depletion (AED)	9.6 (3.3)	9.1 (3.1)	0.194
**Rectal temperature (℃)**			
Baseline	38.7 (1.5)	38.5 (0.9)	0.328
Insult nadir	38.5 (0.5)	38.2 (1.0)	0.442
1–24 h post HI	38.5 (0.2)	38.5 (0.2)	0.232
24–48 h post HI	38.5 (0.3)	38.3 (0.2)	0.234
**Heart rate (min^−1^)**			
Baseline	159 (17)	158 (14)	0.189
Insult nadir	178 (17)	159 (21)	0.955
1–24 h post HI	182 (30)	185 (19)	0.482
24–48 h post HI	165 (19)	184 (20)	0.336
**MABP (mmHg)**			
Baseline	54 (6)	47 (10)	0.189
Insult nadir	35 (10)	31 (5)	0.955
1–24 h post HI	51 (3)	49 (4)	0.482
24–48 h post HI	51 (4)	53 (6)	0.336
**PaO_2_ (kPa)**			
Baseline	12.9 (5.3)	12.2 (4.5)	0.888
Insult nadir	6.1 (1.11)	4.9 (1.0)	0.894
24 h post HI	13.0 (5.45)	18.1 (9.7)	0.161
48 h post HI	26.0 (16.4)	18.6 (4.1)	0.463
**PaCO_2_ (kPa**			
Baseline	4.5 (1.2)	7.2 (2.2)	0.135
Insult nadir	6.1 (1.8)	7.1 (2.9)	0.805
24 h post HI	5.3 (1.9)	5.2 (1.8)	0.798
48 h post HI	7.4 (4.0)	7.3 (5.3)	0.530
**Base excess (mmol/L)**			
Baseline	7.0 (3.2)	3.5 (4.2)	0.130
Insult nadir	−4.7 (3.5)	−6.4 (3.3)	0.259
24 h post HI	2.6 (5.2)	0.5 (4.9)	0.574
48 h post HI	4.6 (4.4)	0.0 (3.9)	0.073
**Blood lactate (mmol/L)**			
Baseline	2.8 (0.7)	3.1 (1.0)	0.328
Insult nadir	7.9 (2.7)	9.3 (2.4)	0.152
24 h post HI	3.6 (1.6)	2.6 (1.7)	0.232
48 h post HI	1.1 (0.5)	1.3 (0.9)	0.731
**Blood pH**			
Baseline	7.47 (0.05)	7.43 (0.06)	0.645
Insult nadir	7.30 (0.22)	7.23 (0.16)	0.521
24 h post HI	7.45 (0.15)	7.45 (0.08)	0.574
48 h post HI	7.37 (0.20)	7.35 (0.22)	0.699
**Haematocrit**			
Baseline	23.4 (4.9)	20.5 (3.7)	0.279
Insult nadir	23.7 (4.5)	22.0 (5.4)	0.382
24 h post HI	19.9 (4.2)	21.8 (3.0)	0.328
48 h post HI	20.3 (3.2)	21.6 (4.0)	0.537

Note: Time zero was set at the time of reperfusion/resuscitation. Mean and standard deviation (SD) values are presented for the two groups; (i) Hypoxia-ischemia (HI) (*n* = 8) and (ii) HI and RIPostC (*n* = 8). Analysis using Mann Whitney test indicated that there was no evidence of a difference between the two groups for any of the outcomes at any of the time-points. Insult severity was estimated by calculating the time integral of the change in NTP/epp during HI and the first 60 min of resuscitation.

**Table 2.** Volume and inotrope requirements.

Treatment	HI	HI & RIPostC
	Median (IQR)	
Volume replacement (ml/kg/h)	0.8 (0.4–1.1)	0.7 (0.6–0.7)
Dopamine (mcg/kg/min)	7.5 (0.0–15.9)	8.3 (4.0–13.3)
Dobutamine (mcg/kg/min)	0.1 (0.0–4.4)	0.5 (0.0–2.4)

Note: Mean (IQR) values for total volume replacement and dopamine and dobutamine infusions for the two groups. Analysis using Kruskal–Wallis equality of populations rank test revealed no evidence of a statistically significant difference between groups for volume replacement and inotrope use.

**Figure 2.**
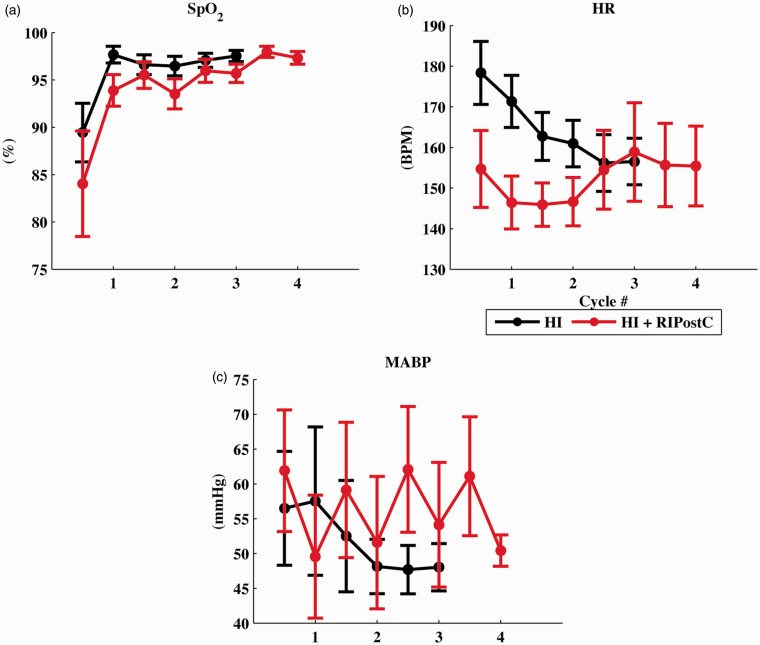
(a) Oxygen saturation (SpO_2_), (b) Heart rate (HR) and (c) Mean arterial blood pressure (MABP) during the four RIPostC limb ischemia/reperfusion cycles. There was no significant difference between groups, although with each RIPostC ischemia/ reperfusion cycle there was a corresponding increase/decrease in MABP.

The continuous physiological measurements (blood oxygen saturation levels_,_ heart rate and mean arterial blood pressure) during the four RIPostC cycles are shown in Figure 2. There was no significant difference between groups in oxygen saturation, HR or MABP during the cycles. An increase and decrease in MABP was visible with each RIPostC ischemic and reperfusion cycle, respectively. There was full recovery of perfusion to the lower limbs following the four RIPostC cycles and no leg injuries observed.

### MRS analysis showed reduced white matter injury in the RIPostC group

**Figure 3.**
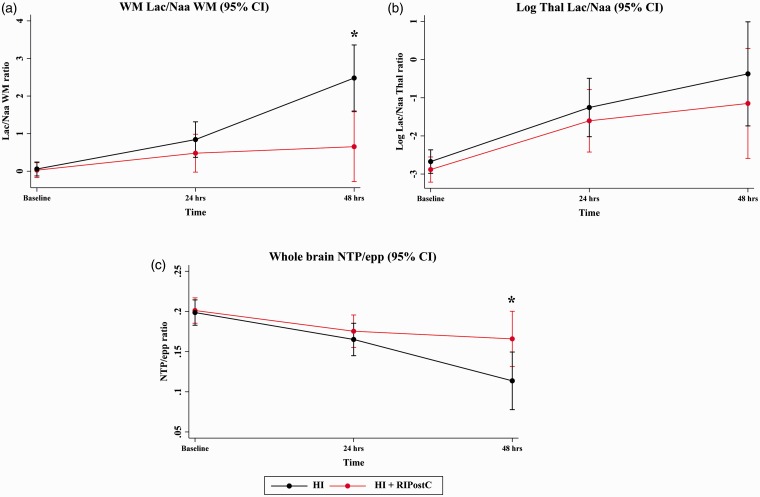
(a–c) Graphs showing metabolite ratios and 95% CIs at baseline, 24 and 48 h from the regression model. (a) WM Lac/Naa, (b) Thalamic Lac/Naa and (c) NTP/epp. **p = 0.005; *p = 0.039. epp = exchangeable phosphate pool; HI = hypoxia–ischemia; Lac = lactate; Naa = N-acetyl aspartate; Thal = thalamic; WM = white matter.

In one piglet in the RIPostC group, ^1^H MRS could not be acquired due to technical problems with the MRI system. One subject in the HI group had a cardiac arrest prior to 48 h; this was related to ventilation failure. ^1^H MRS was used to measure cerebral Lactate/Naa and ^31^P MRS to measure whole brain NTP/epp. Both these metabolite peak area ratios correlate with neurodevelopmental outcome in infants with NE.^13,16^ The differences between the HI and HI + RIPostC groups and associated 95% confidence intervals at each of the three time points (baseline, 24 and 48 h) are shown in Figure 3(a) and Supplementary Table 2. White matter Lactate/Naa was significantly lower in the HI + RIPostC group compared to the HI group at 48 h (*p* = 0.005). NTP/epp was significantly higher at 48 h in the HI + RIPostC group compared to the HI group (*p* = 0.03). Thalamic ^1^H MRS showed no differences between groups at any time point.

### EEG/aEEG findings

The aEEG background voltage at 6 h after birth is predictive of neurodevelopmental outcome in NE^23^ and the rate of aEEG recovery after HI predicts outcome.^24^ All piglets had score 3 (normal background voltage) at baseline. Fitting an ordered logistic regression model to the data, and allowing for repeated measurements over time in each pig, there was no evidence of any difference in the effect of treatment at different time points (*p* = 0.206).

### Histopathology analysis reveals a reduction in cell death in white matter and higher numbers of surviving oligodendrocytes with RIPostC

**Figure 4.**
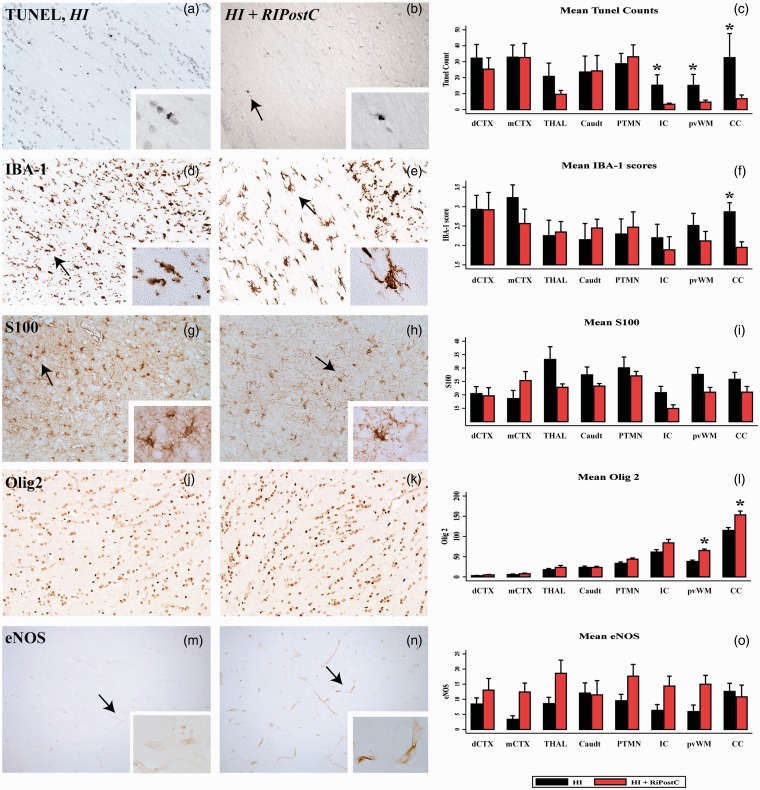
RIPostC decreases histological damage in white matter at 48 h after a hypoxic ischemic insult in the neonatal piglet model. Each column shows representative sections from the same animal in the HI (left column) and HI + RIPostC groups (right column) from the periventricular white matter (pvWM). The tissue is stained for TUNEL at ×20 (a–b) magnification, immunohistochemistry for microglial IBA1 (d–e), S100 (g–h), Olig 2 (j–k) and eNOS (m–n). Mean cell counts are shown in the right column. Compared to no treatment after HI, with RIPostC we found significantly reduced TUNEL-positive cells in the internal capsule, periventricular white matter and corpus callosum (c), reduced IBA-1 scores in the corpus callosum (f) and increased Olig 2 counts in the periventricular white matter and corpus callosum (l). The arrow in (b) indicates a TUNEL positive cell, seen in the inset at ×63 magnification. The arrow in (d) indicates a highly scored microglial cell with loss of secondary processes and short thickened primary with intense soma (score 3), seen in the inset at x40 magnification. The arrow in (e) indicates a low scored microglial cell with tertiary processes, but increased staining intensity of the soma (score 1), seen in the inset at x40 magnification. The arrows in (g) and (h) indicate S100 positive astrocytes. The arrows in (m) and (n) indicate eNOS positive cells. The increase in eNOS in the RIPostC group did not reach significance (o). HI = hypoxia-ischemia; RIPostC = remote ischaemic post conditioning; dCTX = dorsal parietal cortex, mCTX = midtemporal cortex; THAL = thalamus; Caudt = caudate; PTMN = putamen; IC = internal capsule; pvWM = periventricular white matter; CC = corpus callosum.

Levels of cell death and changes in glia were measured using TUNEL staining for fragmented DNA and cell specific markers; Iba1 for microglia, S100B for astrocytes, and Olig2 for oligodendrocytes. The number of TUNEL positive cells observed in the HI + RIPostC versus HI group was significantly reduced in all assessed areas of the white matter: periventricular white matter, internal capsule, and corpus callosum (Figure 4 and Supplementary Table 3). RIPostC had no effect on number of TUNEL positive cell number in the grey matter regions.

Quantification of the numbers of cells expressing Olig2 showed significantly higher number of Olig2 positive cells in the HI + RIPostC group in the periventricular white matter and corpus callosum compared to the HI group (Figure 4). RIPostC increased numbers of cells expressing Olig2 to similar levels as those seen in naïve brain in the corpus callosum, but not in the periventricular white matter (Supplementary Figure 1). We observed no significant difference in levels of astroglial activation (S100B) in the HI + RIPostC group versus HI (Figure 4 and Supplementary Table 3). We observed a significant reduction of microglial activation (Iba1) in the corpus callosum in the HI + RIPostC group (*p* = 0.01) in comparison to HI group (Figure 4 and Supplementary Table 3). There was no significant difference in eNOS expression in the HI + RIPostC group when compared to HI (Figure 4 and Supplementary Table 3).

### Gene expression analysis

**Figure 5.**
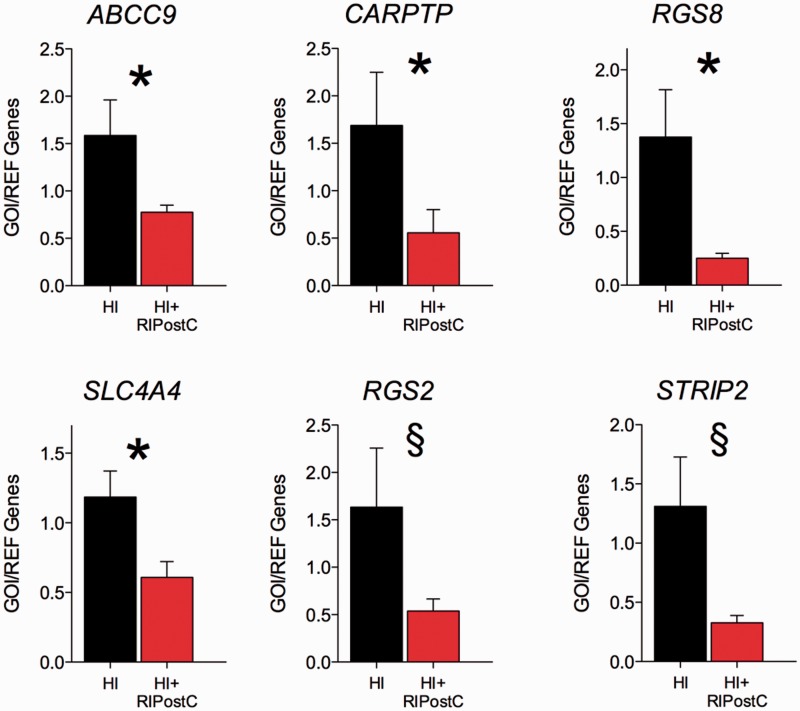
Real-Time qRT-PCR (Real Time quantitative Reverse Transcription PCR) of periventricular white matter tissue in HI and HI + RIPostC groups. In the white matter, compared with HI only, RIPostC had a significantly reduced gene expression for ABCC9 (ATP sensitive potassium channels ATP-binding cassette, sub-family C member 9), CART (cocaine and amphetamine-regulated transcript), RGS8 (Regulator of G-protein signaling-8) and SLC4 (Sodium bicarbonate co-transporter) and trends for reductions (*p* < 0.08) were observed in STRIP2 (striatin Interacting Protein 2) and RGS2.

In the microarray analysis, an initial total of 74 genes were found to be responsive to our RIPostC intervention at the cut off *p* < 0.05. Of these 74 genes, 63 were downregulated and 11 were upregulated. The top 20 identified gene transcripts with the highest fold-change in expression are shown in Table 3; all 20 were downregulated. When the Benjamini-Hochberg FDR multiple testing correction was applied, no candidate gene remained significant. However, we chose to use a targeted approach to further investigate a number of targets with qRT-PCR and these data are shown in Figure 5. We found that, compared with HI only, RIPostC cortical tissue had a significantly reduced gene expression for ABCC9 (ATP sensitive potassium channels ATP-binding cassette, sub-family C member 9), CARPTP (cocaine and amphetamine-regulated transcript), RGS8 (Regulator of G-protein signaling-8), and SLC4 (Sodium bicarbonate co-transporter), and trends for reductions (*p* < 0.08) were observed in STRIP2 (striatin Interacting Protein 2) and RGS2.

**Table 3.** Top 20 identified gene transcripts with the highest fold change in expression by RIPostC.

Gene transcript (Abbreviation)	Gene Identification	Fold Change	P value
Cocaine-and amphetamine-regulated transcript (CARTTP)	NM_001099925	−3.21	0.012
Mitochondrial specific tRNA for aspartate		−2.64	0.021
Protein phosphatase 1 regulatory subunit 1B (PPP1RIB)		−2.47	0.012
Regulator of G-protein signalling 2, 24 kDa (RSG2)	NM_001044600	−2.47	0.009
Matrix Gla protein (GLA)	NM_2141167	−2.35	0.036
Regulator of G-protein signalling 8-like (RSG8)	XM_00357571	−2.11	0.006
UPF0672 protein Chromosome X open reading frame 36 homolog (Cxorf36)	NC_010461	−2.09	0.046
Serglycin (proteoglycan) (Srgn)	NC_010461	−2.03	0.046
ATP-binding cassette, sub-familyC (CFTR/MRP), member 9 (ABCC9)	XM_005655658	−2.00	0.021
Long-chain-fatty-acid–CoA ligase ACSBG1-like Acyl-CoA synthetase bubblegum family member 1 (Acsbg1/ mBG1) (Acsbg1mBG1)	XM_005656246.1	−1.96	0.006
Endothelin receptor type A (EDNRA)	NM_214229.1	−1.96	0.046
Glutamate decarboxylase 2 (pancreatic islets and brain, 65 kDa) (GAD2)	NM_213895	−**1.93**	0.046
Probable G-protein coupled receptor 88-like striatal specific G-protein coupled receptor (Gpr 88STRG)	NC_010454.3	−1.92	0.006
Calcium/calmodulin-dependent 3′,5′-cyclic nucleotide phosphodiesterase1B (PDE1B)	NC_010454.3	−1.92	0.036
Carboxypeptidase M (CPM)		−1.2	0.046
Macrophage scavenger receptor 1 (CD204)	XM_005672635	−1.86	0.046
Solute carrier family 4, sodium bicarbonate cotransporter, member 4 (SLC4A4)	NM_001030533.1	−1.85	0.027
Translocation associated membrane protein 1 (TRAM1L1)	NC_010444	−1.85	0.027
Striatin interacting protein 2 (STRIP2)	XM_003134694	−1.84	0.027
EGF latrophilin and seven transmembrane domain containing 1 (ELTD1)	XM_003127914	−1.83	0.046

Note: Genes were thought to be affected by RIPostC if their expression was affected by more than 1.5 fold with *p* < 0.05.

## Discussion

This study in newborn piglets demonstrates selective protection of cerebral white matter with four 10-minute cycles of RIPostC (bilateral lower limb ischemia/reperfusion) started immediately after a global cerebral HI insult. Immediate RIPostC improved cerebral energy metabolism on localized white matter ^1^H MRS (Lactate/Naa) and increased levels of whole brain cerebral ATP (^31^P MRS NTP/epp). Correlating with improved cerebral energy metabolism in the white matter in the RIPostC group, cell death was reduced in the periventricular white matter, internal capsule, and corpus callosum, and there were higher numbers of oligodendrocytes in the corpus callosum and periventricular white matter. Microglial activation was reduced in the corpus callosum. We did not observe any grey matter protection on ^I^H MRS or immunohistochemistry. We detected changes in expression of a number of genes following RIPostC treatment in the periventricular white matter at 48 h after HI, including the ATP-sensitive potassium channel (K_ATP_) and the endothelin A receptor.

We monitored brain metabolism using MRS biomarkers that are known to correlate with injury severity after HI in the piglet.^14,25^ and outcome in infants with NE.^26^ These biomarkers are currently used as surrogate outcome measures in clinical neuroprotection studies of NE (https://www.npeu.ox.ac.uk/toby-xe). High levels of thalamic Lactate/Naa on MRS in neonates in the first month after birth are predictive of a poor 12–18-month neurodevelopmental outcome.^16,26^ In this study, RIPostC immediately after HI abrogated the white matter MRS lactate/Naa increases at 48 h and preserved whole brain ATP on ^31^P MRS. Higher ATP on ^31^P MRS in infants with NE is associated with better long-term outcome in clinical studies.^13^

The protective mechanisms of RIPostC are incompletely understood, but are thought to involve three closely related pathways (neuronal, humoral and systemic) initiated by the release of endogenous autocoids (including adenosine, bradykinin and opioids) from the skeletal muscle.^27^ The *neuronal* pathway is triggered via stimulation of local afferent nerves, leading to remote protection via efferent nerves including the autonomic nervous system.^28^ The *humoral* pathway involves the circulation of blood-borne protective factors whose release is triggered by the limb ischemia and efferent nerve activation. The *systemic* pathway describes the effects on immune function (including reduced neutrophil activation) and reduced expression of apoptotic and inflammatory genes. The three pathways converge on the brain to increase cerebral blood flow, attenuate inflammation, and activate cellular pro-survival signaling cascades, resulting in mitochondrial protection, reduced energy demands, increased cell survival, and activation of repair mechanisms.

Brain injury after perinatal HI is amplified by both innate and adaptive immune mechanisms. Macrophages responsible for innate immunity can be derived from either residential microglia or monocytes from peripheral blood. We observed a significant reduction in activated microglia in the corpus callosum with RIPostC (Figure 4 and Supplementary Table 3). This is in keeping with other studies, for example in mice, IPostC blocked infiltration of both innate and adaptive immune cells following focal cerebral ischemia and this was associated with reduced neurological deficits.^29^

Our MRS data reflect improved mitochondrial metabolism in the white matter with reduced Lactate/Naa and overall higher whole brain NTP/epp in the RIPostC group. Increased cell survival with RIPostC was seen in the corpus callosum, internal capsule and periventricular white matter, reflected by reduced TUNEL positive cells. RIPostC also increased oligodendrocyte survival in the periventricular white matter and corpus callosum, reflected by increased Olig2 staining. The superior cerebral white versus grey matter protection seen with RIPostC in our piglet model is intriguing. A vascular component to white matter protection from RIPostC has been suggested in a mouse model of vascular cognitive impairment where RIPostC selectively protected white matter integrity, improved cognitive function, and inhibited inflammation and cell death.^30^ Watershed predominant injury results from chronic partial hypoxia or severe global injury and the areas of most damage are the border zones between the major cerebral arteries. These border zones are the areas most susceptible to ischemia from a fall in perfusion pressure. We observed protection in periventricular white matter, suggesting that RIPostC preserved the internal vascular watershed territories. We saw no improvement in the EEG with RIPostC, which supports the absence of cortical protection on immunohistochemistry. Thus, selective protection of the white matter by RIPostC may open up opportunities to combine RIPostC with therapies that target grey matter to provide optimal global protection. Alternatively, RIPostC may augment white matter protection when administered with therapies that benefit white and grey matter such as hypothermia^31^ and melatonin.^21^

RIPostC acts at the cellular level to directly protect the vascular endothelium via K_ATP_ channel-dependent mechanisms.^32^ Vascular endothelial dysfunction triggered by ischemia/reperfusion can promote vasoconstriction and thrombosis thorough loss of endothelium-derived factors such as nitric oxide (NO); conditioning has been shown to preserve endothelial function, increase NO production, and decrease adhesion of neutrophils to endothelial cells.^33^ In our study, although the increases in eNOS with RIPostC did not reach statistical significance (Figure 4 and Supplementary Table 3), it is possible that small changes in NO through Akt activation was one of the mechanisms leading to white matter protection.

The protective signals from RIPostC activate intracellular survival signaling pathways in the brain.^34^ We observed changes caused by RIPostC in several genes previously associated with RIPostC neuroprotection. The opening of the mitochondrial K_ATP_ channels is one of the endpoints of the postconditioning stimulus. We observed that a transcript required for both the formation of ATP-sensitive potassium channels and the cardioprotective effects of pre- and post-conditioning, the ATP-binding cassette, sub-family C (CFTR/MRP), member 9 (ABCC9) was down-regulated (−2 fold using both microarray and qRT-PCR). The endothelin receptor-A, another gene transcript required for pre-conditioning, was down-regulated (−1.96 fold microarray, −3.00 fold qRT-PCR) and expression of its putative receptor ligand CARPTP was also reduced (−3.21 fold microarray and −3.00 fold qRT-PCR). RIPostC also induced a reduction in gene expression for two genes involved in the negative regulation of G protein-coupled receptor (GPCR) signal transduction pathways, RG8 which inhibits M1 muscarinic acetyl choline GPCR receptor signaling which is required to induce post-conditioning (−2.11 fold microarray, −3 fold qRT-PCR) and RGS2 which is a negative regulator of Angiotensin II GPCR signaling (−2.47 fold microarray, −5 fold qRT-PCR). These data are complex as we are unable to assess changes in expression at multiple time points; at 48 h, some signaling pathways involved in RIPostC mediated neuroprotection may be subjected to negative feedback.

RIPostC was applied to both hind limbs immediately after the onset of cerebral reperfusion by a remotely controlled external device placed on the inguinal crease. We chose the RIPostC stimulus with four cycles of 10-min ischemia/reperfusion in both hind limbs. Ten minutes ischemia was optimal as longer periods of ischemia up to 15 min led to irreversible limb damage in our pilot studies. Both hind limbs were used for the stimulus to increase the muscle mass affected and to optimize the protective response. A recent clinical trial showed better protection with two cycles of leg ischemia than three cycles in arm,^35^ suggesting that the mass of the ischemic muscle is an important factor for organ protection. We used a custom-made remotely controlled external device (Patent: SMK/LP6981443) placed over the inguinal area. There was full recovery of perfusion to the lower limbs following the four RIPostC cycles and no leg injuries observed.

Our study has some limitations. We used only female piglets to minimize the effect of isoflurane conditioning described in male piglets.^36^ This differs from our previous studies where male piglets were used.^21,31^ The more debilitating effect of hypoxia-ischemia on outcomes in males versus females is well known, although in a recent rodent study there was no sex difference in the neuropathology scores.^37^ RIPostC will require assessment in male piglets to ensure benefit is maintained. We were required to use fentanyl for sedation in both experimental groups throughout the experiment due to ethical requirements. A previous RIPostC study in neonatal rat pups demonstrated protection against neonatal hypoxic-ischemic brain injury through activation of the opioid receptor pathway;^11^ because of the fentanyl use, we could not assess these pathways in this study. The gene studies were exploratory; the single 48 h may be subjected to negative feedback and therefore mask evolving signaling pathways involved in RIPostC mediated neuroprotection.

Postconditioning is a potent strategy that can modify reperfusion-induced adverse effects that include defense against oxidants, pro-inflammatory cytokines, neutrophils, and pro-apoptotic regulators and so encompasses a broad therapeutic approach.^6^ Most clinical applications are in myocardial injury; for example, postconditioning improves revascularization following myocardial infarction.^6^ Huge advances have been made in the last 10 years regarding our understanding of mechanisms and potential use in the clinical arena; a workshop in 2014 outlined the importance of preconditioning in acute renal and liver disease as well as in stroke and various cardiac interventions.^38^ In adult stroke, a recent study of 443 adults who underwent pre-hospital remote ischemic per-conditioning as an adjunct to thrombolysis for acute ischemic stroke found a reduced risk of tissue infarction in the treatment group.^39^ Remote ischemic conditioning was safe and well tolerated.

This study shows cerebral white matter protection with four cycles of limb RIPostC started immediately after a global cerebral hypoxic ischemic injury in female newborn piglets. We demonstrate white matter protection using established brain MRS markers of injury and immunohistochemistry. RIPostC influenced the expression of a number of genes thought to mediate the effects of pre/post conditioning. It will be important to assess the therapeutic window of RIPostC and whether RIPostC augments therapeutic hypothermia in pre-clinical studies of perinatal hypoxia-ischemia before studies in babies could be considered. Other challenges include the determination of the optimal number and duration of ischemia/reperfusion cycles while avoiding any detrimental effects and a better understanding of the precise protective mechanisms.^40^

## Supplementary Material

Supplementary material
