# Supplementary material for: Immediate remote ischemic postconditioning after hypoxia ischemia in piglets protects cerebral white matter but not grey matter
Source: J Cereb Blood Flow Metab. 2015 Oct 8;36(8):1396–411. doi: 10.1177/0271678X15608862 (PMC4976661; doi:10.1177/0271678X15608862)
Supplement: Supplementary material [file Supplementary_Material.docx]

**Supplementary Figure 1**


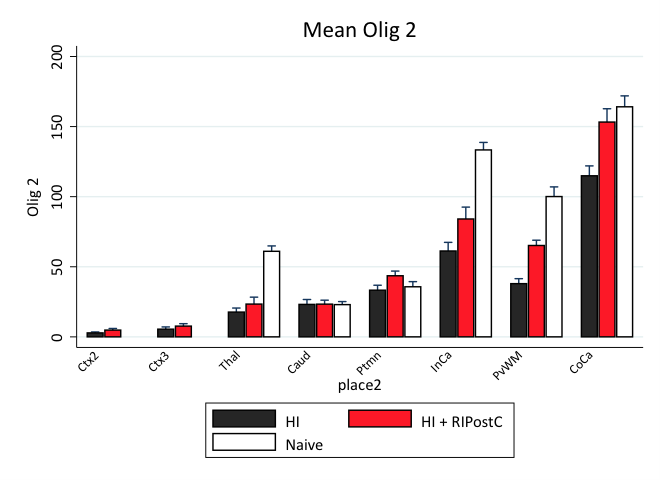


Statistical analysis (linear regression accounting for multiple field measurements) comparing our Naïve group with the RIPostC group showed no significant difference between the mean Olig 2 values in the naïve group and the mean Olig 2 values in the RIPostC group (p=0.637) in the Corpus Callosum, indicating that the effect of post-conditioning was to return the Olig 2 values to Naïve levels. In the PvWM, the mean Olig 2 values were returned someway towards Naïve levels with RIPostC, but remained significantly lower (p<0.001)

HI = hypoxia-ischemia; RIPostC = remote ischaemic post conditioning; dCTX = dorsal parietal cortex, mCTX = midtemporal cortex; THAL = thalamus; Caudt = caudate; PTMN = putamen; IC = internal capsule; pvWM = periventricular white matter; CC = corpus callosum.

**Supplementary Table 1**

| **Gene name** | **Sequence** | | **Product size (bp)** |
| --- | --- | --- | --- |
| *CARTPT* | F | TGCAGGAAGTCCTGAAGAAG | 140 |
|  | R | GGGACAGTCACACAGCTTCC |  |
| *PPP1R1B* | F | AGGAGGATGACGAAGAAGAGG | 100 |
|  | R | CCTTGGCCACAAGTTGTCTT |  |
| *RGS2* | F | CAAAAGCTGTCCTCCAAAGC | 141 |
|  | R | GCAGCCACTTGTAGCCTCTT |  |
| *MGP* | F | GCAGCCCTGTGCTATGAATC | 149 |
|  | R | GGAGGCTTGTTGAGTTCTCG |  |
| *RGS8* | F | AACTGCAAAGCTGGTCTCCA | 99 |
|  | R | GCTTCTCGGGTCTGGAAGTC |  |
| *SRGN* | F | CCTCCAAGGACTGACCCTTT | 121 |
|  | R | CATTTAGGGAGCCACTTCCA |  |
| *ABCC9* | F | ACATCAAACCTGGGCAAAAG | 143 |
|  | R | CAGGGGCAGTTTTGAAATGT |  |
| *EDNRA* | F | TTTCCTGCTCCTCATGGACT | 140 |
|  | R | TGGACTGGTAACAGCAGCAG |  |
| *CPM* | F | GCAGTCATGAAGTGGCTGAA | 145 |
|  | R | CATCATCATCGGGGGTTAAG |  |
| *SLC4A4* | F | AAGGGGAGTCTGGACAGTGA | 131 |
|  | R | TCTCTGTCGGAAGGTTTGCT |  |
| *STRIP2* | F | TGGAGGAAAAGCAACATGAA | 130 |
|  | R | AGGTGCATTCTTCTGCTTGG |  |

Primers designed using the Sus scrofa Ensembl database

**Supplementary Table 2**

|  | **Baseline difference (95% CI)** | **p-value** | **24 hour difference (95% CI)** | **p-value** | **48 hour difference (95% CI)** | **p-value** |
| --- | --- | --- | --- | --- | --- | --- |
| **WM Lac/Naa** | 0.03  (-0.24, 0.30) | 0.8 | 0.36  (-0.33, 1.05) | 0.3 | 1.83  (0.55, 3.11) | 0.005 |
| **Thalamus Lac/Naa** | 0.209  (0.242,0.659) | 0.4 | 0.348  (-0.772, 1.468) | 0.5 | 0.776  (-1.207, 2.759) | 0.4 |
| **NTP/epp** | -0.003  (-0.025,0.020) | 0.8 | -0.010  (-0.039, 0 .018) | 0.5 | -0.052  (-0.102, -0.003) | 0.039 |

Differences between the HI and HI & RIPostC groups and associated 95% confidence intervals at each of the three time points (baseline, 24 and 48 h). These differences between groups were estimated from the model for lactate/Naa in the white matter and thalamus and NTP/epp in the whole brain over the 48 hours. There was a significant difference between groups for white matter lactate/Naa with a lower value at 48 hours in the HI+ RIPostC versus HI group (p= 0.005). There was a significantly higher NTP/epp at 48h in the HI+ RIPostC versus HI group (p=0.039). There was no difference between groups for thalamic lactate/Naa.

**Supplementary Table 3**

|  | **Brain Region** | **Coefficient** | **95% CI lower boundary** | **95% CI upper boundary** | **P value** |
| --- | --- | --- | --- | --- | --- |
| **TUNEL** | Dorsal parietal cortex | -0.34 | -1.11 | 0.43 | 0.389 |
|  | Midtemporal cortex | -0.12 | -0.88 | 0.64 | 0.754 |
|  | Thalamus | -0.46 | -1.18 | 0.27 | 0.215 |
|  | Caudate | -0.001 | -0.71 | 0.71 | 0.998 |
|  | Putamen | 0.05 | -0.68 | 0.79 | 0.885 |
|  | **Periventricular white matter** | **-0.81** | **-1.552** | **-0.06** | **0.03** |
|  | **Internal Cap** | **-1.13** | **-1.85** | **-0.41** | **0.002** |
|  | **Corpus Callosum** | **-0.85** | **-1.57** | **-0.13** | **0.021** |
|  |  |  |  |  |  |
| **IBA-1** | Dorsal parietal cortex | -0.01 | -1.14 | 1.12 | 0.742 |
|  | Midtemporal cortex | -0.67 | -1.64 | 0.31 | 0.181 |
|  | Thalamus | 0.09 | -0.85 | 1.04 | 0.845 |
|  | Caudate | 0.30 | -0.63 | 1.24 | 0.526 |
|  | Putamen | 0.18 | -0.91 | 1.26 | 0.749 |
|  | Periventricular white matter | -0.40 | -1.18 | 0.39 | 0.321 |
|  | Internal Cap | -0.31 | -1.26 | 0.64 | 0.519 |
|  | **Corpus Callosum** | **-0.92** | **-1.46** | **-0.38** | **0.001** |
|  |  |  |  |  |  |
| **S100** | Dorsal parietal cortex | -0.20 | -0.90 | 0.51 | 0.585 |
|  | Midtemporal cortex | 0.03 | -0.59 | 0.66 | 0.923 |
|  | Thalamus | -0.16 | -0.69 | 0.37 | 0.544 |
|  | Caudate | -0.05 | -0.43 | 0.33 | 0.789 |
|  | Putamen | 0.04 | -0.42 | 0.50 | 0.873 |
|  | Periventricular white matter | -0.28 | -0.68 | 0.13 | 0.176 |
|  | Internal Cap | -0.30 | -0.72 | 0.12 | 0.161 |
|  | Corpus Callosum | -0.24 | -0.76 | 0.27 | 0.357 |
|  |  |  |  |  |  |
| **Olig 2** | Dorsal parietal cortex | 1.34 | 0.14 | 12.74 | 0.801 |
|  | Midtemporal cortex | 1.57 | 0.23 | 10.94 | 0.648 |
|  | Thalamus | 1.14 | 0.59 | 2.17 | 0.697 |
|  | Caudate | 1.40 | 0.50 | 3.91 | 0.516 |
|  | Putamen | 1.36 | 0.96 | 1.93 | 0.081 |
|  | **Periventricular white matter** | **1.84** | **1.30** | **2.60** | **0.001** |
|  | Internal Cap | 1.35 | 0.84 | 2.17 | 0.210 |
|  | **Corpus Callosum** | **1.31** | **1.03** | **1.68** | **0.029** |
|  |  |  |  |  |  |
| **eNOS** | Dorsal parietal cortex | 4.61 | -10.11 | 19.33 | 0.539 |
|  | Midtemporal cortex | 9.03 | -2.31 | 20.37 | 0.119 |
|  | Thalamus | 10.05 | -7.05 | 27.14 | 0.249 |
|  | Caudate | -0.58 | -21.14 | 19.99 | 0.956 |
|  | Putamen | 7.72 | -6.94 | 22.38 | 0.302 |
|  | Periventricular white matter | 9.07 | -3.71 | 21.84 | 0.164 |
|  | Internal Cap | 8.06 | -5.47 | 21.60 | 0.243 |
|  | Corpus Callosum | -1.50 | -17.88 | 14.87 | 0.857 |
|  |  |  |  |  |  |

Estimated RIPostC treatment group coefficient with 95% confidence interval and p-value for TUNEL, IBA-1, S100, Olig 2 and eNOS for brain regions specified.
